# Unveiling the roles of Sertoli cells lineage differentiation in reproductive development and disorders: a review

**DOI:** 10.3389/fendo.2024.1357594

**Published:** 2024-04-18

**Authors:** Yang Gao, Zican Wang, Yue Long, Lici Yang, Yongjian Jiang, Dongyu Ding, Baojian Teng, Min Chen, Jinxiang Yuan, Fei Gao

**Affiliations:** ^1^ College of Basic Medicine, Jining Medical University, Jining, Shandong, China; ^2^ State Key Laboratory of Stem Cell and Reproductive Biology, Institute of Zoology, Chinese Academy of Sciences, Beijing, China; ^3^ The Collaborative Innovation Center, Jining Medical University, Jining, Shandong, China; ^4^ Lin He’s Academician Workstation of New Medicine and Clinical Translation, Jining Medical University, Jining, Shandong, China

**Keywords:** Sertoli cell, sex determination, mice, spermatogenesis, embryonic cell development, primordial germ cell

## Abstract

In mammals, gonadal somatic cell lineage differentiation determines the development of the bipotential gonad into either the ovary or testis. Sertoli cells, the only somatic cells in the spermatogenic tubules, support spermatogenesis during gonadal development. During embryonic Sertoli cell lineage differentiation, relevant genes, including *WT1*, *GATA4*, *SRY*, *SOX9*, *AMH*, *PTGDS*, *SF1*, and *DMRT1*, are expressed at specific times and in specific locations to ensure the correct differentiation of the embryo toward the male phenotype. The dysregulated development of Sertoli cells leads to gonadal malformations and male fertility disorders. Nevertheless, the molecular pathways underlying the embryonic origin of Sertoli cells remain elusive. By reviewing recent advances in research on embryonic Sertoli cell genesis and its key regulators, this review provides novel insights into sex determination in male mammals as well as the molecular mechanisms underlying the genealogical differentiation of Sertoli cells in the male reproductive ridge.

## 1 Introduction

In mammals, sex determination is the process by which the reproductive ridges develop into ovaries or testes. The genital ridge, a bipotential structure functioning as a precursor to the gonads, comprises germ cells surrounded by the coelomic epithelium (1). The directed migration of primordial germ cells (PGCs) to the genital ridge is critical for reproductive system development (2). However, the fate of PGCs that reach the genital ridge and differentiate into oogonia or spermatogonia is determined by the surrounding embryonic somatic cells (3). As early as 1967, Ohno proposed that in higher vertebrates, gonadal sexual differentiation is independent of the germ cells themselves and that the fate of the gonads is dominated by steroid hormone-producing molecules (4). Based on this perspective, in 1972, Krzysztof Boczkowski proposed that the main difference between male and female gonadal differentiation in mammals lies in the different mechanisms of gonadal differentiation and development in males and females. According to Boczkowski, the most important step in the process of male differentiation is the development of mesenchymal cells and hormone secretion (5). Palmer and Burgoyne’s 1991 study confirmed that Sertoli cells play a central role in testicular determination (6). Since then, research has been primarily focused on the regulatory mechanisms of Sertoli cell lineage differentiation in the process of sex determination. Somatic and germ cells are both present in the fetal gonads, and the process of sex determination is dominated by somatic cells, which contribute to the formation of the testes or ovaries through spatiotemporally defined expression of relevant genes. Sertoli cells play a key role in testicular determination and gonadal development (7, 8). Sexual differentiation refers to the development of any physical or behavioral characteristics that are unique to either males or females (9). Two duct systems exist in mammals, Wolffian ducts and Müllerian ducts, which eventually develop into male and female ducts, respectively (9, 10). During sex differentiation, one of the duct systems degenerates while the other continues to develop into the corresponding duct system (9, 10). In males, the Müllerian duct degenerates under the influence of AMH secreted by testicular Sertoli cells, and the Wolffian duct develops into the adult male reproductive tract under the action of androgens produced by Leydig cells (9, 10). Disorders of sex development (DSD) are linked to alterations in the development and functionality of Sertoli cells (11). In people with 46, XY, DSD, the development and function of Sertoli cells is dysfunctional, resulting in reduced levels of AMH, which prevents the Müllerian duct from degenerating into the uterus and fallopian tubes (11). This gender development disorder is known as persistent müllerian duct syndrome (PMDS). Two morphologically distinct Leydig subpopulations, fetal (FLC) and adult Leydig cells (ALC), are present during testicular development (12, 13). In the testis, the first wave of cells entering the gonad produces Sertoli cells that later promote the differentiation of steroidogenic cells entering the gonad into Leydig cells (14). Leydig cells are present in the testicular interstitial compartment and regulate male fertility. FLC population formation has multiple cell origins, including the somatic epithelium (15). Among them, FLC progenitor cells originating from the somatic epithelium gradually lose Notch activation upon entry into the fetal testis and thus differentiate directly into FLC (16). The number of FLCs increases until the fetus is born, peaks at birth, and then decreases gradually (13, 17). During pubertal testicular maturation, ALC gradually replace FLC (13, 18). After male puberty, Sertoli cells are the major somatic cell component in the testis and directly regulate the proliferation and differentiation of germ cells, support the typical spermathecal structure, and induce germ cells to ensure male spermatogenic output (19–22). The timing of Sertoli cell differentiation ultimately determines the number of Sertoli cells and the upper limit of male fecundity (23, 24). USF proteins (including USF1 and USF2) and cell-division control protein 42 (CDC42) regulate Sertoli cell proliferation and differentiation, thereby affecting the final number of Sertoli cells in the testis and imposing limits on fertility (25, 26). In addition, downregulation of Tetraspanin 8, Sostdc1, and miR-92a-3p in pubertal Sertoli cells results in is the maintenance of normal sperm quantity and quality (27, 28). In this process, downregulation of miR-92a-3p is induced by gonadotropins, and gonadotropin promotes the proliferation of immature Sertoli cells and targets Sertoli cells to support the expansion and differentiation of pre-meiotic germ cells (28–30). Several theories have been proposed on the mechanism underlying the origin of Sertoli cells; however, further studies are warranted to explore their embryonic origin. An increasing number of studies have reported on the molecular mechanisms of gonadal differentiation and the associated regulatory factors. Nonetheless, most studies are based on relatively independent studies of one or a few nodal factors and lack a systematic analysis linking the many regulatory factors. For this reason, in this review, we provide a systematic account of the molecular mechanisms of the numerous regulatory genes that contribute to the lineage differentiation process of Sertoli cells and the correlations among them. In this review, we discuss the mechanisms involved in embryonic Sertoli cell development, explore the gene defects associated with Sertoli cell development in DSD models, and highlight the critical factors in Sertoli cell lineage differentiation, providing an updated research perspective on the diagnosis and treatment of DSD and related diseases in the early embryonic stage.

## 2 Genital ridge formation in mammals

In mouse embryos, genital ridge formation begins around 10.5 days *post coitum* (dpc), continuing until 11.5–12.0 dpc. This developmental period aligns with the beginning of gonadal sex differentiation (31, 32). Concurrently, at approximately 9.5 dpc, the ventral epithelial layer of the mesonephros thickens in pairs, causing the basement membrane to protrude and disintegrate. Within the coelomic epithelium, certain cells proliferate and differentiate into the mesenchyme, migrating toward the dorsomedial mesenchymal region. These morphological alterations initially manifest at the cephalic end before gradually extending caudally to form the genital ridge (33).

PGCs originate from the embryonic ectoderm and migrate to reach the genital ridge (34) at approximately 10.5 dpc (35). Upon arrival, PGCs are enveloped by somatic cells derived from the coelomic epithelium. Steroidogenic factor-1 (SF1) is an essential transcription factor for genital ridge formation and development. In mice, *SF1* expression is first detected in the urogenital ridge at 9 dpc (36), localizes to the adrenal primordium at 11 dpc, and concentrates in the adrenal cortex at 13 dpc (37). Its pattern of expression in humans is similar (38, 39). SF1 deficiency disrupts the testicular pathway, leading to ectopic ovarian development and potentially causing partial or complete inversion of the male-female gonadal system post-birth (40). In XY sex-determining region Y-box 9 (*SOX9)*-*CRE*-*SF1* cKO mice at 12.5–13.5 dpc, testicular marker levels are significantly decreased, whereas ovarian marker levels are increased. At 10.0 dpc, GATA binding protein 4 (GATA4) is a genital ridge (genetic ridge) formation marker that is expressed only in somatic cells and is required for the initiation of genital ridge formation (32). Previously, it was widely believed that germ cell licensing occurred independently of gonadal somatic cells (41–43). However, Hu et al. showed that the PGC transition to meiotic germ cells is likely to depend on gonadal somatic cells and that systemic knockout of *GATA4* prevents somatic epithelial proliferation in the somatic lumen, leading to rupture of the basement membrane and the absence of germinal ridges, thus causing germline cells to remain at the PGC stage, ultimately losing the ability to differentiate between sexes (32, 44). In addition, GATA4 also promotes *SF1* expression in progenitor cells (44).

The expression of several genes is necessary for the growth and maintenance of the genital ridge, including LIM Homeobox 9 (*LHX9*), Wilms’ Tumor 1 (*WT1*), and Empty Spiracles Homeobox 2 (*EMX2*) (32). LHX9 is involved in a variety of developmental processes, including gonadogenesis. During sex differentiation, LHX9 upregulates *SF1* and promotes the proliferation of somatic epithelial cells and disintegration of the basement membrane, thereby contributing to the formation of the germinal ridge and proliferation of somatic cells in the germinal crest (45, 46). Mice with a conditional knockout of *LHX9* in the gonads display normal germ cell migration with reduced *SF1* expression, while somatic cells in the germinal ridge are unable to proliferate and form discrete gonads (46). WT1 plays a critical role in lineage specification and the maintenance of gonadal somatic cells and acts as a transcription factor required for somatic cell survival in the genital ridge. It is initially expressed in the mesoderm of the 9 dpc genital ridge region and subsequently in the somatic epithelium. In addition, it could potentially be expressed in the mesenchymal cells of the urogenital tract ridges at 9.5 dpc (34). Deletion of *WT1* results in embryonic mortality, apoptosis of genital ridge somatic cells, and metanephros defects. In contrast, a lack of *EMX2* impedes the migration of epithelial cells through the basement membrane (47). After these processes, sex-determining gene expression is initiated, which determines the sex and fate of its germ cells as the bipotential genital ridge differentiates into either a testis or an ovary (33, 45).

## 3 Role of Sertoli cell lineage differentiation in male sex determination

The sex-determining region Y (SRY) on the Y chromosome, which controls the development of a male phenotype, is key to determining mammal sex. If *SRY* is expressed, testes form and most male secondary sexual characteristics develop under the influence of hormones secreted by the testes. GATA4 is a critical transcription factor required for *SRY* expression in Sertoli progenitor cells (48). GATA4 binds to the *SRY* promoter in a mitogen-activated protein kinase (MAPK)-dependent manner *in vivo*, thereby ensuring the timely expression of *SRY* (49, 50). SRY acts as a switch that prompts the development of the germinal ridge into the testis. During embryogenesis, *SRY* expression triggers the differentiation of Sertoli cells into Sertoli progenitor cells that would otherwise form granulosa cells in the ovary. Following *SRY* expression, several testis-specific events are elicited, including the development of a testis-specific vascular system.

During mouse embryonic development between 10.5 and 12.5 dpc, when the genital ridge remains undifferentiated, *SRY* is temporarily expressed in XY embryos, which peaks at approximately 11.5 dpc (51). Consequently, SOX9 is rapidly translocated from the cytoplasm to the nucleus. SRY, along with SF1, activates *SOX9* expression (52) by binding to the testicular enhancer core (TESCO) sequence. *SOX9* expression triggers a cascade of transcriptional events and promotes testicular formation (53, 54). SRY uses both cell-autonomous mechanisms and prostaglandin D2 (PGD2)-mediated signaling mechanisms to stimulate *SOX9* expression and induce the differentiation of Sertoli cells *in vivo* (55).

Activated SOX9 activates *SRY* expression (54). Once a certain threshold is reached, SOX9 inhibits *SRY* expression and promotes its expression by interacting with SF1 (56). If either SRY or SOX9 activation fails to occur, the development of granulosa cells and ovarian pathways is initiated instead (57, 58). The expression of *SOX9* also enhances the expression of testicular development-related factors, such as prostaglandin D2 synthase (*PTGDS*), anti-Müllerian hormone (*AMH*), *WT1*, *GATA4*, and ex-determining region Y-box 8 (*SOX8*), while concurrently inhibiting the expression of ovarian determinants, including *WNT4*, R-spondin 1 (*RSPO1*), and *β-catenin* (51, 54, 59). Moreover, SOX9 can further enhance its own expression by activating the PGD2 and fibroblast growth factor 9 (FGF9) signaling pathways (60), thereby forming a positive feedback loop (51).

During testicular development, FGF9 plays a critical role in two distinct steps (61). Initially, it stimulates the proliferation of cell populations that generate Sertoli progenitor cells (61). Within these progenitor cells, FGF9 facilitates the nuclear localization of fibroblast growth factor receptor 2 (FGFR2) (62). In mice, FGF9/FGFR2 signaling inhibits WNT4, antagonizes female development by repressing female-promoting genes, and drives testicular differentiation (63, 64). *FGFR2* expression in Sertoli cells coincides with *SRY* expression and aligns with the male developmental markers (*FGF9*-activated receptors) of *SOX9* downstream of *SRY* (61), suggesting that FGFR2 has a mediating role in Sertoli progenitor cells across various stages of testicular development (62). In mice, the deletion of *FGFR2* in the embryonic gonad is identical to that of *FGF9* and indicates sex reversal from male to female (62). Furthermore, knocking out either *FGF9* or its receptor *FGFR2* leads to significant changes in the somatic niche, including reduced *AMH* expression, fewer *SOX9*-positive Sertoli cells, and alterations in the spermatic cord structure, including decreased *AMH* expression, decreased number of *SOX9*-positive Sertoli cells, and altered spermatic cord structure (65). Activation of the PI3K/AKT signaling pathway promotes proliferation and prevents apoptosis in immature Sertoli cells (66, 67). In immature Sertoli cells, FGF binds to its receptor FGFR and activates *PI3K* expression (68). PI3K phosphorylates PIP2 to produce PIP3 (66), which then activates AKT1 by directly inducing AKT1 phosphorylation or activating PDK1 and mTORC2 to phosphorylate different sites on AKT1 (66). Consequently, AKT activates the expression of its downstream gene *mTORC1* (66), which in turn activates the expression of *p70S6K* and *EMI1* in the nucleus (69). rpS6, a substrate of p70S6K, facilitates MYC protein expression, thereby upregulating Cyclin A1/2 expression (66, 70). EMI1 maintains SKP2 levels by inhibiting SKP2 degradation, which upregulates Cyclin D1/3-CDK4, Cyclin E1/3, and PCNA, ultimately promoting the proliferation and survival of immature Sertoli cells (66).

**Figure 1:**
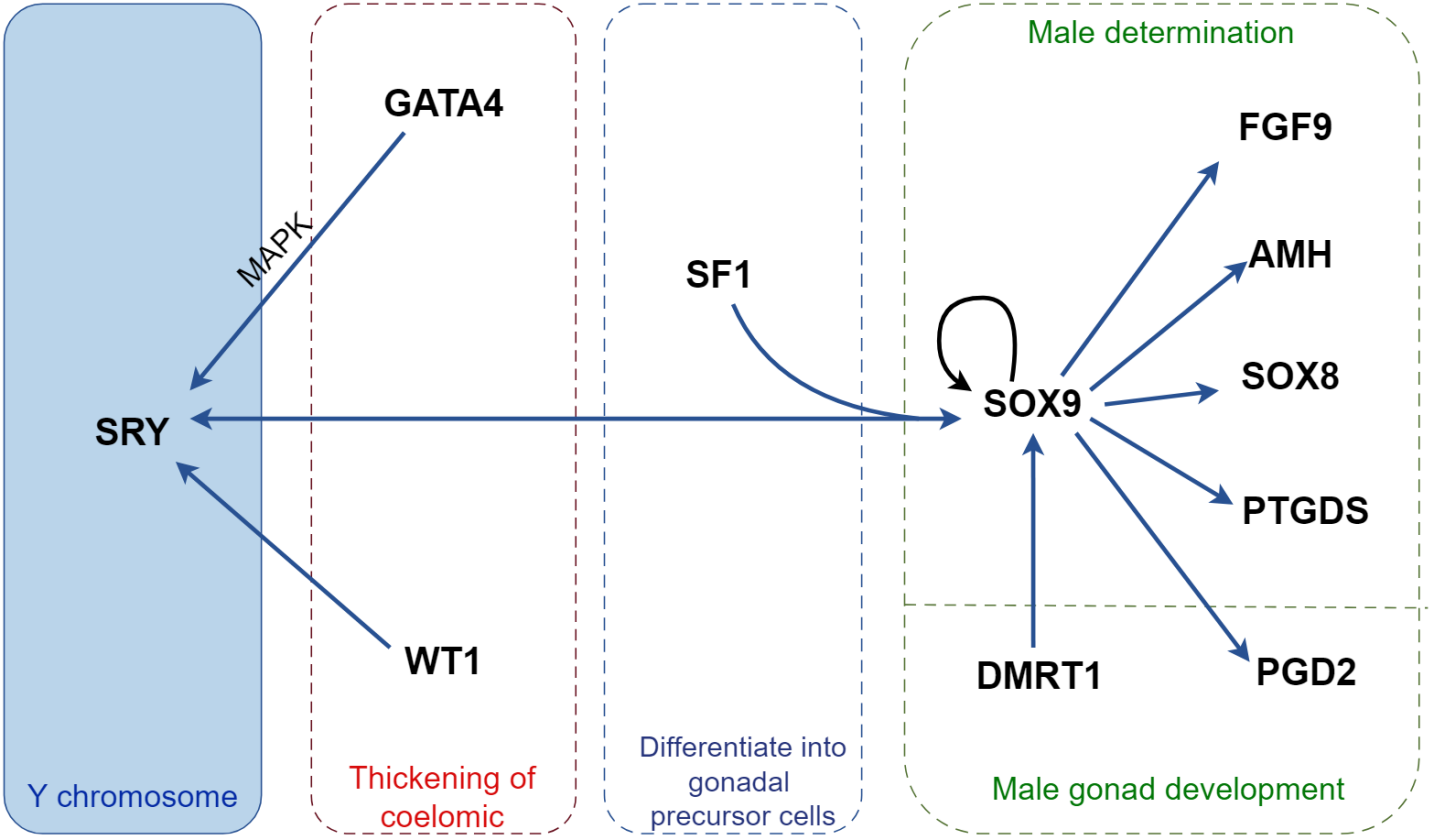
Regulatory mechanisms of cytokines in male sex determination in mice. *SRY*, the only sex-determining gene located on the Y chromosome, binds to *GATA4* in a MAPK-dependent manner to facilitate its own on-time expression and cooperates with *SF1* to promote *SOX9* expression. Activated *SOX9* induces *SRY* expression and forms a positive feedback pathway with *WT1* and *DMRT1* to promote further *SOX9* expression. *SOX9* promotes the expression of testis development-associated factors, including *FGF9*, *AMH*, *SOX8*, *PTGDS*, and *PGD2*, facilitating embryonic development into a male phenotype. MAPK, mitogen-activated protein kinase; SOX9, sex-determining region Y-box 9; AMH, anti-Müllerian hormone; DMRT1, doublesex and mab-3 related transcription factor 1; FGF9, fibroblast growth factor 9; GATA4, GATA binding protein 4; PGD2, prostaglandin D2; PTGDS, prostaglandin D synthase; SF1, steroidogenic factor-1; SRY, sex-determining region Y; WT1, Wilms’ tumor 1.

Doublesex and mab-3-related transcription factor 1 (*DMRT1*) expression is triggered at 12.5 dpc, promoting the proliferation of Sertoli cells and maintaining male characteristics (71). DMRT1 inhibits the expression of *WNT4* and Forkhead box L2 (*FOXL2*), and its overexpression transcriptionally activates the expression of *PTGDS*, *FGFR2*, and glial cell-derived neurotrophic factor (*GDNF*). In mice with conditionally deleted DMRT1, Sertoli cells transdifferentiate into granulosa cells, and the ovaries undergo recombination (72) (**Figure 1**; **Table 1**).

**Table 1:** Cytokines involved in Sertoli cell lineage differentiation.

Gene name	Upstream gene	Downstream gene	Location of expression	Function
*WT1*	—	*SF1*, *GATA4*, *AMH*, *DHH*	Coelomic epithelium of the urogenital ridge and lower Leydig cells	Guiding the fate of common progenitor cells, determining whether they become Sertoli cells or fetal Leydig cells
*SF1*	*WT1*, *GATA4*, *LHX9*	*SOX9*, *SRY*	Testis	Initiating gonadal and testicular differentiation, contributing to mammalian sex determination
*SOX9*	*SF1*, *SRY*	*DMRT1*, *FGF9*, *PTGDS*, *PGD2*	Testis	Involved in the early specification of male Sertoli cells
*DMRT1*	*SOX9*	*PGD2*	Genital ridge	Male gonadal fate maintenance

AMH, anti-Müllerian hormone; DHH, desert hedgehog; DMRT1, doublesex and mab-3 related transcription factor 1; FGF9, fibroblast growth factor 9; GATA4, GATA binding protein 4; LHX9, LIM homeobox 9; PGD2, prostaglandin D2; PTGDS, prostaglandin D synthase; SF1, steroidogenic factor-1; SOX9, sex-determining region Y-box 9; SRY, sex-determining region Y; WT1, Wilms’ tumor 1.

Between 11.5 and 12.5 dpc, cells of the male gonads form testicular cords after undergoing extensive cellular rearrangements (73). The reorganization of Sertoli cells, the vascular system, and Leydig cells simultaneously orchestrate testicular cord morphogenesis. Sertoli cells drive testicular morphogenesis under the guidance of the vascular system (74, 75). In mice, somatic vasculature and interstitial microvasculature in the male gonad are formed by vascular endothelial cells migrating from the mesonephros during testis formation (74, 76, 77). Vascular endothelial cell-mesenchymal interactions determine the fate of testicular cord formation (74, 78, 79). Furthermore, macrophages are present in the fetal testis and promote neoplastic morphogenesis of the testicular cord by controlling vascularization and tissue pruning, as well as removing somatic cells and errant germ cells after testicular structures are established (80).

## 4 Abnormalities in the molecular regulation of Sertoli cell lineage differentiation are involved in DSD: insights into genetic contributions

During mammalian embryogenesis, Sertoli cells play a vital role in the determination of the testes and the comprehensive development of the gonads, acting as the central components of testicular functionality. However, in patients with DSD, the typical development and function of Sertoli cells are often disrupted. To date, over 60 genes have been associated with 46,XY DSD (81).

A study identified 57 mutations across nine genes in 56 patients with 46,XY DSD (82). In approximately 60% of the patients, a variant of *AR*, *SRD5A2*, or *SF1* was found to cause 46,XY DSD (82). Recently, seven patients carrying mutations in genes known to be pathogenic for 46,XY DSD were identified; four of these mutations were in *DHX37*, two in *MYRF*, and one in *PPP2R3C* (82). Furthermore, *HSD17B3*, *SRD5A2*, and *AR* are associated with androgen synthesis and action, and *PPP2R3C* and *MYRF* are linked to syndromic 46,XY DSD, whereas *SRY*, *DHX37*, *GATA4*, and *SF1* are related to testicular determination and development (82). Despite these insights, the genetic etiology of >50% of DSD patients remain unclear, implying the possible existence of numerous unidentified sex-determining genes. As a result, we will dissect the key regulators controlling Sertoli cell lineage differentiation during embryonic development and their mechanisms of action one by one.

WT1, SF1, SOX9, and DMRT1 play major roles in embryonic development. WT1 promotes the lineage differentiation of Sertoli cells through the non-canonical Wnt/planar cell polarity (PCP) pathway and the expression of downstream *SF1* by acting as an upstream factor (83). SF1 participates in the differentiation of the Sertoli cells along with WT1, initiating gonadal and testicular differentiation and promoting the expression of its downstream gene *SOX9* (84). SOX9 plays a role in the early specification of Sertoli cells and maturation of testicular cords and promotes the expression of the downstream gene *DMRT1*. DMRT1 influences Sertoli cell maturation and polarity and maintains the Sertoli cell phenotype in postnatal testes. In addition, WT1, SF1, SOX9, and DMRT1 interact during embryonic development, influence the genealogy and differentiation of Sertoli cells, and promote embryonic sex differentiation.

### 4.1 WT1

**Figure 2:**
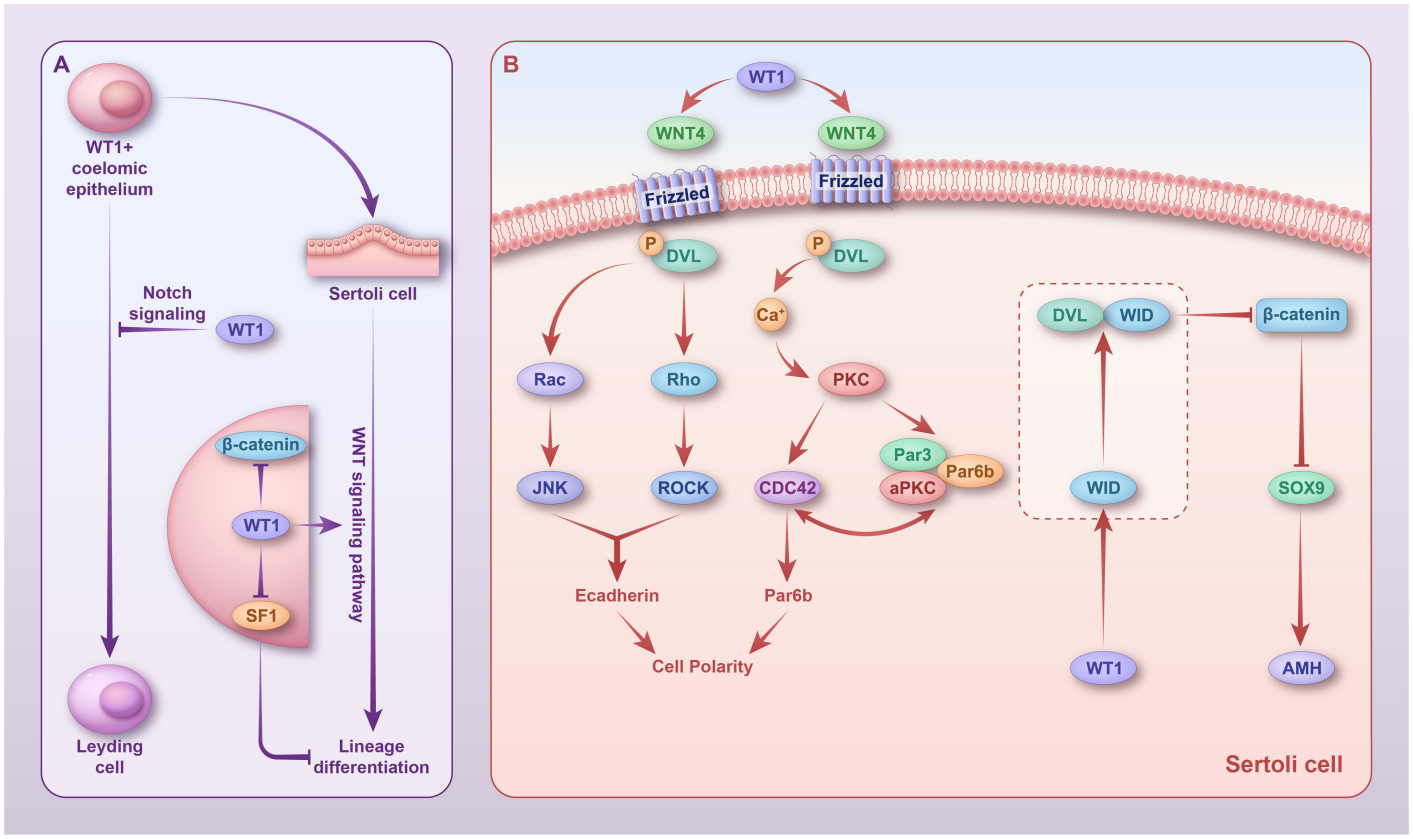
Regulatory mechanisms of *WT1* in sex differentiation. **(A)** In fetal *WT1* ^+^ somatic cells, *WT1* inhibits their differentiation into Leydig cells by activating the Notch pathway. In Sertoli cells, *WT1* persists and promotes the lineage differentiation of Sertoli cells by inhibiting *SF1* and activating the non-canonical Wnt pathway. **(B)** In Sertoli cells, *WT1* activates *WNT4*, which activates two non-canonical Wnt pathways. In the Wnt/PCP signaling pathway, phosphorylated *DVL* activates Rac and Rho; Rac1 activates JNK; and *RHO* activates the related helix protein ROCK. JNK and ROCK then work together to regulate cell polarity. In the Wnt/Ca^+^ signaling pathway, Ca^2+^ activates PKC, which further stimulates *CDC42* and promotes actin polymerization, thereby affecting cell polarity. *WT1* promotes the expression of its target gene, *WID*, which forms a complex with *DVL* to inhibit *WNT3a* expression and, thus, β-catenin signaling. However, whether this mechanism exists in Sertoli cells remains to be confirmed. WT1, Wilms’ tumor 1; DVL, phosphorylated Disheveled.

During embryonic development, *WT1* is expressed in the somatic epithelial and inferior Leydig cells of the urogenital ridge (85). It has been postulated to influence the destiny of *WT1* ^+^ somatic cells in the testes, directing their differentiation into either Sertoli cells or FLC (71) (**Figure 2A**). Moreover, the presence of *WT1* in pro-Sertoli cells fosters a bias toward Sertoli cell differentiation (71). Its overexpression in the adrenal gland inhibits the development of steroidogenic cells (86), thereby counteracting the expression of steroidogenesis-related enzymes and modulating the balance between the mesenchyme and the epithelium, as well as the Sertoli cell lineage. Our findings suggested that *WT1* deficiency can trigger the transformation of Sertoli cells into Leydig cells (87). In contrast, *WT1* overexpression in Leydig cells induces the expression of Sertoli cell-specific genes while inhibiting steroidogenic genes (87).

*WT1* regulates Sertoli cell polarity via the activation of two non-canonical Wnt signaling pathways, the Wnt-PCP signaling pathway and the Wnt/Ca^+^ signaling pathway, thereby promoting lineage differentiation and spermatogenesis of Sertoli cells (83) (**Figure 2B**). In the Wnt/PCP signaling pathway, phosphorylated Disheveled (*DVL*) activates Rac and Rho, Rac1 activates Jun N-terminal kinase (JNK), and Ras homolog gene-family (*RHO*) activates the related helical protein ROCK. Together, JNK and ROCK regulate cell polarity. In the Wnt/Ca^+^ signaling pathway, Ca^2+^ activates protein kinase C (PKC), which in turn stimulates *CDC42*, thereby promoting actin polymerization and affecting cell polarity (88). Using a conditional knockout *WT1* mouse model, we found that *WT1* inactivation resulted in massive spermatogenic cell death in adult mice, with only Sertoli cells present in most seminiferous tubules (89).

Histological studies have revealed that *WT1*-deficient testes have compromised blood-testis barrier integrity (89). Knockdown of endogenous *WT1* in neonatal calf Sertoli cells using RNAi technology does not impact the proliferative capacity of Sertoli cells but rather downregulates genes associated with cell polarity (par-6 family cell polarity regulator beta and E-cadherin) and Wnt signaling (*WNT4* and *WNT11*) (90).

β-Catenin is an important effector of the canonical Wnt signaling pathway and a regulator of cell adhesion; hence, its inhibition during embryonic development is critical for testicular development (91). During testis development, *WT1* inhibits β-catenin signaling in Sertoli cells; thus, its deletion increases β-catenin expression in Sertoli cells, both *in vitro* and *in vivo* (91). In addition, *WT1* promotes the expression of its target gene, *WID*, which forms a complex with *DVL* to inhibit *WNT3a* expression (92) and, thus, β-catenin signaling. Whether this mechanism exists in Sertoli cells remains unclear. Although the classical Wnt/β-catenin signaling pathway is not involved in the lineage differentiation of Sertoli cells, it has been shown to affect spermatogenesis in adult mice (93). The presence of β-catenin in Sertoli cells maintains testicular development; however, activation of the Wnt/β-catenin signaling pathway leads to defective spermatogenesis. *WT1* inhibits the activation of the Wnt/β-catenin signaling pathway in the testis, ensuring normal spermatogenesis. In mice, the expression of the stabilized form of β-catenin in Sertoli cells disrupts the germ cell microenvironment, leading to failure of spermatogenesis (94). In humans, abnormal accumulation of β-catenin in Leydig cells can lead to spermatogenesis failure, leading to non-obstructive azoospermia and infertility (95).

*WT1* regulates the development of FLC and Leydig progenitor cell lineage via Notch signaling and inhibits *WT1* ^+^ Leydig cell differentiation into FLC (96, 97). A subpopulation of WT1^+^ progenitor cells differentiated into Sertoli cells upon *SOX9* expression, whereas WT1^+^ cells lacking SOX9 differentiated into Leydig cells of the testis (71). In mesenchymal progenitor cells, *WT1* promotes the expression of the Hes family bHLH transcription factor 1 (*HES1*) and jagged canonical Notch ligand 1 (*JAG1*) (96) by promoting the expression of *NOTCH2* and *NOTCH3* (97). *HES1* prevents mesenchymal progenitor cells from differentiating into FLC by inhibiting the transcription of the cell cycle inhibitor gene cyclin-dependent kinase inhibitor 1B (*CDKN1B*) (98, 99). Hedgehog signaling that occurs after Sertoli cell differentiation, promotes the expression of GLI1 in HES1^+^ Leydig cell progenitors (71). Eventually, Leydig cells develop into steroid-producing embryonic mesenchymal or non-steroidogenic cells. Notably, the population of steroid-producing embryonic Leydig cells is limited by Notch2 signaling from neighboring cells, and non-steroidogenic progenitor cells give rise to adult mesenchymal cells after puberty (71). In addition, Notch signaling can maintain the expression of its progenitor cell markers, such as *LHX9* and *NES* (96). Specifically, deleting *WT1* in Sertoli cells at 14.5 dpc during fetal testis development shifts the FLC differentiation state from Leydig progenitors (97). This deletion results in structural disruption of the testis, persistence of FLC-like clusters, failure of adult Leydig cell development, loss of gene expression associated with Leydig cell development (100), and, ultimately, male sterility (101). In addition, *WT1* influences Sertoli cell lineage differentiation by suppressing *SF1* expression. We have previously reported that *WT1* directly binds to the promoter region and suppresses *SF1* expression (102). Remarkably, *WT1* deletion before sex determination upregulates *SF1* expression and impedes the differentiation of supporting cells (102). In pre-pubertal testicular development, *WT1* interacts with heterogeneous nuclear ribonucleoprotein U (*HNRNPU*) in Sertoli cells, binds to endogenous *WT1*, and activates the transcription function of *WT1* on its target genes (103), thereby maintaining the testicular microenvironment. *HNRNPU* knockout leads to testicular atrophy in mice (101).

### 4.2 SF1

**Figure 3:**
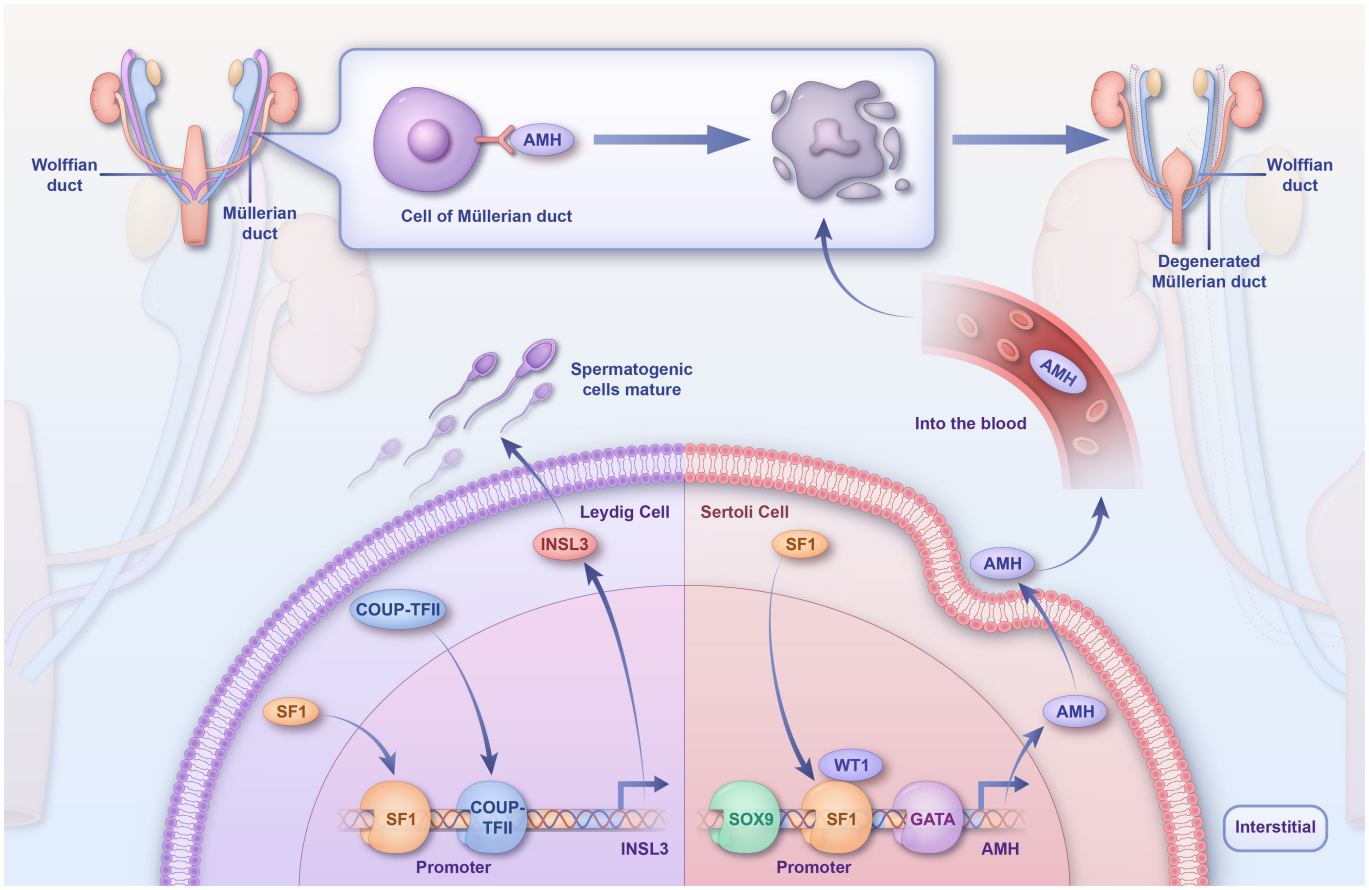
Regulatory mechanisms of *SF1* in sex differentiation. *SF1* directly binds *TES*, which synergistically activates TESCO with *SRY*, leading to a rapid upregulation of *SOX9*. *SF1* then binds to *TES* along with *SOX9* to maintain *SOX9* expression in Sertoli cells. *SF1* activates the *AMH* promoter *in vivo* and inhibits the production of Müllerian tubules, and COUP-TFII and *SF1* synergistically activate the *INSL3* promoter, which promotes spermatogonial cell development and maturation. TES, testis-specific enhancer of SOX9; TESCO, testicular enhancer core; SF1, steroidogenic factor-1; *SOX9*, sex-determining region Y-box 9.

In the developing gonad, SF1 initiates gonadal and testicular differentiation in conjunction with several other transcription factors, including WT1, GATA, EMX2, and SOX9 (84) (**Figure 3**). Furthermore, researchers have proposed that WT1 might be essential for controlling basal level *SF1* expression in the undifferentiated genital ridge and antagonizing *SF1* expression in post-sex determination Sertoli cells (102).

At 9 dpc, *SF1* is first expressed in the gonadal primordium and later persists in the bisexual gonads (54). Moreover, selectively disrupting *SF1* in mice impedes gonadal development (84) and induces rupture of the spermatic cord. In conditional knockout *SF1* mice, the levels of testicular markers decreased, whereas those of ovarian markers increased between 12.5 and 13.5 dpc (40). By 18.5 dpc, the number of Sertoli and spermatogenic cells decreased considerably, with only a marginal spermatic cord structure remaining (104). In mice testes, Sertoli cells delay maturation (105). These findings highlight the fact that *SF1* deletion interrupts the testicular pathway and stimulates ectopic ovarian pathways, leading to partial or complete male-female gonadal inversion post-birth.

During embryonic testis development, *SF1* activates the promoter of anti-Müllerian hormone (AMH) in Sertoli cells and is directly involved in sex determination in mammals (106). Deleting *SF1* in Sertoli cells results in fewer androgen receptors (58, 105). Persistent Müllerian duct syndrome (PMDS) is a 46,XY DSD usually caused by mutations in *AMH* or a single-base deletion in the *SF1* response element. In patients with PMDS, a regulatory mutation has been identified as a single-base deletion in the *SF1* response element of the *AMH* promoter, significantly diminishing its *SF1*-binding capacity (107).

In Leydig cells, *SF1* regulates the transcription of genes involved in testosterone synthesis and sterol biosynthetic enzymes. Leydig cells secrete 90% of the testosterone in males, which promotes the development and maturation of the sex organs and spermatogenic cells. Dihydrotestosterone, which is catalyzed from testosterone by the enzyme 5α-reductase, leads to prostate and penile growth and fusion of the labial folds (sexual differentiation) (108). Testosterone is synthesized by the steroidogenic enzyme Cyp11a1, which converts cholesterol to pregnenolone. Pregnenolone is then further converted to testosterone by Hsd3b, Cyp17a1, and Hsd17b3 (108). *SF1* is involved in testosterone biosynthesis by regulating the transcription of sterol biosynthesis; in Leydig cells, COUP-TFII and SF1 physically interact (109). Insulin-like 3 (INSL3) is a Leydig cell-specific hormone, which, during fetal life, is essential for normal male sex differentiation and testicular descent during the transabdominal period (110). The INSL3 promoter contains species-conserved binding sites for COUP-TFII (-91 bp) and *SF1* (-134 bp) (111); hence, COUP-TFII and *SF1* can synergistically activate the INSL3 promoter.

SF1 regulates the MDM2/TP53 pathway during development and is pivotal for the survival of fetal Sertoli cells (104). p53, an oncoprotein encoded by *TP53*, is a transcription factor that regulates the initiation of the cell cycle by controlling the expression of specific genes. SF1 activates p53 by directly regulating *MDM2* expression through direct binding to the P1 promoter of *MDM2* (112).

### 4.3 SOX9

In the mammalian testes, SOX9 is critical for the initial differentiation of male Sertoli cells. HNRNPU interacts with SOX9 and enhances the expression of the transcription factors *SOX8* and *SOX9* in Sertoli cells through direct binding to the promoter regions (101). In both humans and mice, the loss of *SOX9* expression leads to the XY embryo developing into a female. Conversely, erroneous activation of *SOX9* leads to the embryo developing into a male (60). Furthermore, deletion of *SOX9* in XY gonads interferes with the activation of the male-specific markers Mis and P450scc and induces the expression of female-specific markers such as bone morphogenetic protein 2 and follicle-stimulating hormone. SOX9 also influences Sertoli cell differentiation and affects the activation of *SOX8* through tissue-specific knockdown methods (113). Animal studies have shown that WT1 can directly or indirectly regulate *SOX9* after the cessation of *SRY* expression. The expression of the testis-determining gene *SOX9* is switched off in Sertoli cells after *WT1* resection at 14.5 days post-conception (114).

*SOX9* is continuously expressed in the testes throughout fetal development. During sex determination, SRY upregulates *SOX9* expression in undifferentiated embryonic gonads, thereby promoting the differentiation of coelomic epithelium cells into Sertoli cells (115). Despite the ability of SRY to directly or indirectly activate *SOX9*, it is not necessarily required for the sustained expression of *SOX9*. *SOX9* was found to be responsible for the rapid disappearance of *SRY* expression at approximately 12.5 dpc, and *SRY* expression persisted in XY gonads lacking SOX9 (55). AMH inhibits the expression of Müllerian ductal development. Moreover, *SOX9*-positive pre-Sertoli cells express *AMH* and align with spermatogenic cells into primitive cords at 12.5 dpc, which then rapidly transform into more mature testicular cords by 13.5 dpc (116).

The transcription factors SRY and SOX9, along with the RSPO1/WNT4/β-catenin signaling pathway, are involved in an antagonistic pathway for testicular and ovarian development in the mouse embryo, driving the development of common gonadal primordia (117). Rspo1, Wnt4, and β-catenin inhibit testicular cord formation, and targeted deletion of *Rspo1* in mice prevents Wnt4 upregulation while triggering the proliferation of androgen-producing cells (118). In the XY gonad, a positive feedback loop between Sox9 and Fgf9 (as well as PGD2) is established to suppress Wnt4/Rspo1 expression in a paracrine manner, thereby promoting Sertoli cell differentiation (118). Using mouse models with double knockouts of *SOX9* and *WNT4*, researchers have found that SOX9, rather than the inhibition of signaling by RSPO1/WNT4/CTNNB1, is vital for the early determination of male Sertoli cells in the testes (117).

**Figure 4:**
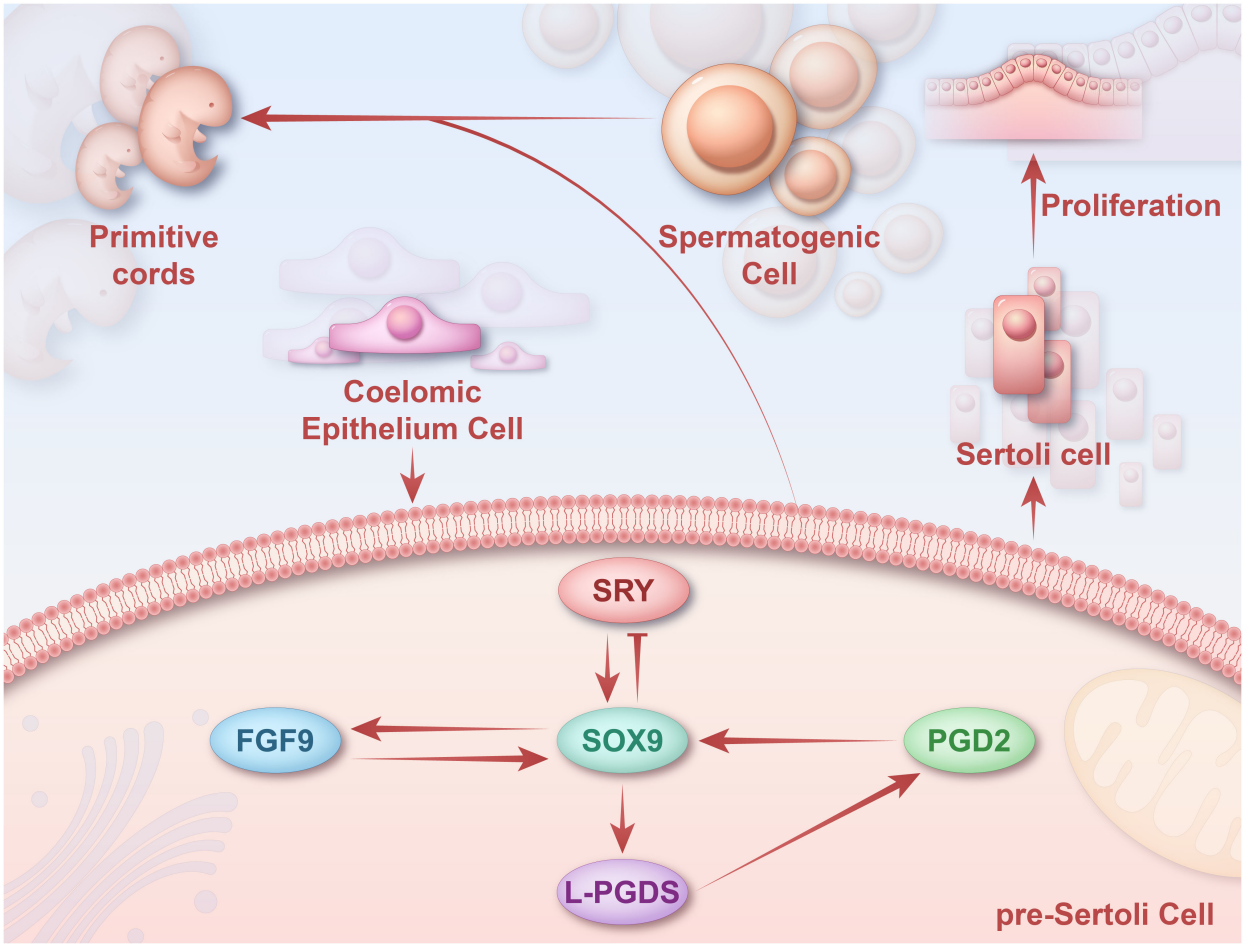
Regulatory mechanisms of *SOX9* in sex differentiation. SOX9 can maintain *SOX9* expression in Sertoli cells through three pathways mediated by *SF1*, *FGF9/FGFR2*, and *PGTDS/PGD2*. *SRY* upregulates *SOX9* expression in undifferentiated gonads by binding to *TES*. SOX9 upregulates *FGF9* and forms a positive feed-forward loop with *FGF9* to maintain *SOX9* expression. *SOX9* promotes the expression of *PTGDS in vivo* by binding to the *PTGDS* promoter. The persistence of SOX9 promotes the production of L-PGDS, which leads to the accumulation of PGD2 and thus activates the transcription of *SOX9*. Later, these *SOX9* ^+^ pre-Sertoli cells will form the primitive cords with spermatogenic cells. SOX9, sex-determining region Y-box 9; SRY, sex-determining region Y; FGF9, fibroblast growth factor 9; FGFR2, fibroblast growth factor receptor 2.

Sertoli cells can coordinate the development of all other male-specific cell types. For proper testicular development, it is critical for Sertoli cells to differentiate in adequate numbers, necessitating sustained and sufficient expression of *SOX9*. At least three mechanisms can effectively maintain the expression of *SOX9* in Sertoli cells (**Figure 4**). In Sertoli cells, SF1 promotes and maintains *SOX9* expression. In precursor support cells, SRY and SF1 directly bind to the testis-specific enhancer of *SOX9* (*TES*) and synergistically activate TESCO, leading to rapid upregulation of *SOX9* (56, 113, 119, 120). Once SOX9 reaches a critical threshold, SRY is repressed through a *SOX9*-dependent negative feedback loop. After *SRY* expression ceases, SF1 binds to enhancers along with SOX9, regulating itself to retain high levels of expression in Sertoli cells (56, 113, 119, 120). The absence of TESCO or *TES* results in a 60% or 45% decrease in *SOX9* expression in XY fetal gonads, respectively, and both have been shown to decrease *AMH* expression (121). Early testes produce PGD2, which induces the differentiation of pre-Sertoli cells into Sertoli cells. *In vivo*, SOX9 forms a binding association with the *PTGDS* promoter and is promptly expressed in Sertoli cells (122). Activation and the continuous presence of SOX9 result in the production of testicular Lipocalin-type PGTDS, leading to the accumulation of PGD2. Subsequently, PGD2 activates the transcription process and aids in the nuclear translocation of *SOX9* (123). *PGTDS* and *SOX9* continue to be expressed in *FGFR2* mutant follicles, suggesting that the prostaglandin pathway maintains *SOX9* expression independently of *FGFR2* (124).

In XY gonads, SOX9 and FGF9 form a positive feed-forward loop essential for somatic cell proliferation. SOX9 is responsible for the expression of *FGF9*, and FGF9 is necessary to sustain the expression of *SOX9* (125). In male XY mouse gonads, SOX9 upregulates *FGF9*, initiating the *SOX9*/*FGF9* feed-forward loop (123). Moreover, SOX9 can enhance *SRY* expression by activating the *FGF9* and *PGD2* signaling pathways (60).

DSD is caused by either the deletion or overexpression of *SOX9*, arising from mutations in the *SOX9* coding region or misregulation or disruption of the regulatory region (126). In two patients with 46,XX testicular DSD, an 83.8 kb repeat was detected in the region 600 kb upstream of *SOX9* (127). Rearrangements within or around *SOX9* can be detected in some patients using chromosome microarray.

In postnatal Sertoli cells, CCAAT enhancer binding protein beta activates *SOX9* expression. cAMP-responsive element binding protein 1 is recruited to the proximal promoter region of *SOX9* through the cAMP/protein kinase A signaling pathway, leading to CREB1 phosphorylation, which promotes *SOX9* expression (128).

### 4.4 DMRT1

*DMRT1* mRNA initially appears in the germinal ridge of mice at approximately 10.5 dpc, with its abundance significantly increasing by approximately 14.5 dpc. DMRT1 is critical for the maturation and polarity of Sertoli cells. *In vivo* studies have demonstrated incomplete maturation of Sertoli cells in males lacking *DMRT1*. Immunohistochemical analyses of the polarity markers ESPIN and NECTIN-2 revealed that DMRT1 is necessary for *NECTIN-2* expression in Sertoli cells (129). DMRT1 cooperates with SOX9 to maintain and reprogram sexual cell fates. Remarkably, DMRT1 appears to play the role of a pioneer transcription factor that binds to otherwise “closed” and inaccessible chromatin and facilitates its opening, thereby creating conditions for the binding of other regulators, including SOX9 (130, 131). DMRT1 binds to DNA in a very unusual way and can bind according to different stoichiometries (131).

DMRT1 is essential for sex determination and preservation of the Sertoli cell phenotype in postnatal testes. In mammals, DMRT1 is required to sustain the male gonadal fate (132). Induction of *DMRT1* expression in the fetal gonads of XX mice using the *WT1*-BAC transgenic system is sufficient to stimulate testicular differentiation and foster the development of male secondary sexual characteristics (133). Deletion of *DMRT1* results in the sex reversal of mice to female at birth. In male mice, deletion of *DMRT1* activates FOXL2 and reprograms Sertoli cells into granular cells, which form follicular cells that produce estrogen, ultimately leading to germ cell feminization (72). The successful nuclear import of *DMRT1*, along with its interaction with importin-β1, ensures the nuclear retention of *DMRT1* and its subsequent influence on the downstream targets of the sexual development cascade (134). *DMRT1* inhibition changes the expression of pivotal genes involved in gonadal development, ultimately leading to focal testicular hypoplasia in the human fetus (135).

DMRT1 is crucial in regulating sperm differentiation (136); its deletion in spermatogonia inhibits the cyclic gene expression in Sertoli cells (137). DMRT1 promotes migration and differentiation of mitotic PGCs (early PGCs) to mitotic-blocking PGCs (late PGCs), and prevents premature meiotic initiation by inhibiting retinoic acid signaling and Stra8 expression (138, 139). At the mouse pubertal stage, DMRT1 is able to activate GDNF signaling and interacts with PLZF, which in turn upregulates Sohlh1 expression, thus participating in the process of spermatogonia self-renewal and differentiation (140). In addition, DMRT1 promotes the expression of the receptor tyrosine kinase (RTK) signaling pathway inhibitory protein *Spry1*. SPRY1 binds to nuclear factor κB1 (NF-κB1), which prevents p65 nuclear translocation, inhibits the activation of the NF-κB signaling pathway, and protects the integrity of the BTB, thereby creating a stable environment for smooth spermatogenesis (141).

Low *DMRT1* expression in mouse testes decreases spermatogonia and destabilizes the testicular ecological niche *in vivo*. The interaction between DMRT1 and promyelocytic leukemia zinc finger (PLZF) proteins is crucial for maintaining male germline stem cell self-renewal (142).

## 5 Summary conclusion and future direction

**Figure 5:**
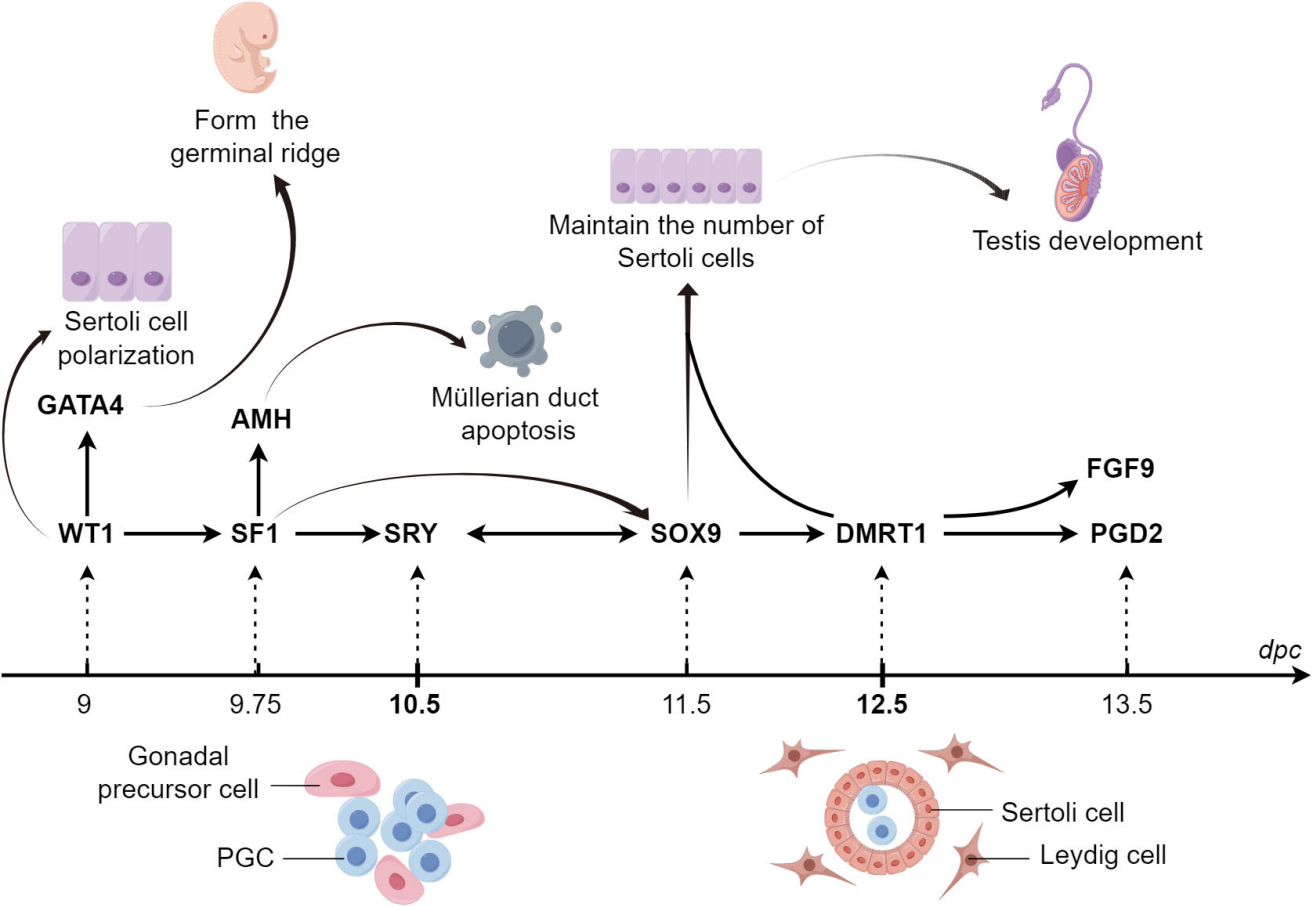
Graphical summary During sex determination in the male embryo, the expression of numerous cytokines at specific times and spaces act together to regulate the lineage differentiation of Sertoli cells. GATA4 ensures the transition of PGCs to meiotic germ cells by promoting the formation of germinal ridges. WT1 influences the polarity of Sertoli cells through the regulation of the non-canonical WNT pathway, which promotes the lineage differentiation of Sertoli cells. SF1 participates in mammalian sex determination through the activation of the promoter of AMH. SOX9 maintains its expression in Sertoli cells through three pathways mediated by SF1, FGF9/FGFR2, and PGDS/PGD2, respectively, thus ensuring that a sufficient number of Sertoli cells are available to induce testicular development. GATA4, GATA binding protein 4; PGCs, primordial germ cells; SF1, steroidogenic factor-1; AMH, anti-Müllerian hormone; *SOX9*, sex-determining region Y-box 9; FGF9, fibroblast growth factor 9; PGD2, prostaglandin D2; PTGDS, prostaglandin D synthase.

Numerous cytokines orchestrate the differentiation of the Sertoli cell lineage during embryonic development. GATA4 ensures the transition of PGCs to meiotic germ cells by promoting the formation of germinal ridges. WT1 affects the polarity of Sertoli cells by regulating the non-canonical Wnt pathway, thereby promoting their lineage differentiation. SF1 is involved in mammalian sex determination by activating the AMH promoter. SOX9 maintains its expression in Sertoli cells through three pathways mediated by SF1, FGF9/FGFR2, and PGTDS/PGD2, which ensures that sufficient Sertoli cells induce testicular development. DMRT1 and SOX9 cooperatively maintain Sertoli cell proliferation; DMRT1 deletion results in male-to-female sex reversal (**Figure 5**).

Despite extensive research spanning decades, substantial gaps remain in our understanding of gonadal sex determination, with many vital aspects yet to be fully elucidated. A comprehensive understanding of gonadal development and differentiation depends on the thorough characterization of all cell types involved in this bipotential primordium and the understanding of their interrelations. Moreover, the molecular factors that regulate the various cell lineages emerging from this process remain largely unknown. Although WT1 reportedly negatively regulates the Wnt/β-catenin pathway through its target gene *WID* in the kidney, whether this mechanism also operates in Sertoli cells to ensure normal spermatogenesis warrants further investigation. In humans, mutations or deletions in the short arm of chromosome 9, where *DMRT1* is located, result in varying degrees of sex reversal during the embryonic period. However, mice with inhibited *DMRT1* expression are born male and only undergo sex reversal postnatally. Further research is required to pinpoint the causes of these discrepancies.

The development of advanced sequencing technologies, such as single-cell RNA sequencing, not only addresses this need but also provides the field of developmental biology with a unique opportunity to identify previously uncharacterized cell types and rare lineages. Furthermore, a comprehensive understanding of the role of cytokines in Sertoli cell lineage differentiation is vital to elucidate the intricate mechanisms underlying testicular development and sex determination. Further investigation into the precise molecular interactions and signaling pathways involved in cytokine-mediated Sertoli cell differentiation will provide insights into the potential therapeutic targets for sexual development and infertility disorders. In conclusion, in this review, we explored the recent breakthroughs in the development of Sertoli cells and their pivotal regulators to both deepen our understanding of sex determination in male mammals and illuminate the intricate molecular mechanisms governing the differentiation of Sertoli cells within the male reproductive ridge. Nevertheless, it is important to acknowledge the limitations of this review. First, the primary focus of this review was to elucidate the roles of specific factors of particular importance. As a result, we did not provide a comprehensive and in-depth analysis of other related factors. Additionally, due to space constraints, we could only describe the roles of associated factors within specific signaling pathways without delving into the broader implications and relevance of these signaling pathways.

## Author contributions

YG: Writing – review & editing, Writing – original draft. ZW: Writing – review & editing. YL: Writing – review & editing. LY: Writing – review & editing. YJ: Writing – review & editing. DD: Writing – review & editing. BT: Writing – review & editing. MC: Writing – review & editing. JY: Writing – review & editing. FG: Writing – review & editing, Writing – original draft.
